# Evolution Driven Microscale Combinatorial Chemistry in Intracellular Mimicking Droplets to Engineer Thermostable RNA for Cellular Imaging

**DOI:** 10.1002/smll.202409911

**Published:** 2025-01-26

**Authors:** Andrew Brian Kinghorn, Wei Guo, Lin Wang, Matthew Yuk Heng Tang, Fang Wang, Simon Chi‐Chin Shiu, Kwan Kiu Lau, Chandra Jinata, Aditi Dey Poonam, Ho Cheung Shum, Julian Alexander Tanner

**Affiliations:** ^1^ School of Biomedical Sciences LKS Faculty of Medicine The University of Hong Kong Hong Kong China; ^2^ Department of Mechanical Engineering Faculty of Engineering The University of Hong Kong Hong Kong China; ^3^ Advanced Biomedical Instrumentation Centre Hong Kong Science Park, Shatin, New Territories Hong Kong China; ^4^ Faculty of Health and Environmental Engineering Shenzhen Technology University 3002 Lantian Road Shenzhen Guangdong 518118 China; ^5^ Department of Chemistry and Department of Biomedical Engineering City University of Hong Kong Hong Kong China; ^6^ Materials Innovation Institute for Life Sciences and Energy (MILES) HKU‐SIRI Shenzhen Guangdong 518063 China

**Keywords:** aptamer, fluorescence, live cell imaging, microfluidic, thermostable

## Abstract

Fluorescent light‐up aptamer/fluorogen pairs are powerful tools for tracking RNA in the cell, however limitations in thermostability and fluorescence intensity exist. Current in vitro selection techniques struggle to mimic complex intracellular environments, limiting in vivo biomolecule functionality. Taking inspiration from microenvironment‐dependent RNA folding observed in cells and organelle‐mimicking droplets, an efficient system is created that uses microscale heated water droplets to simulate intracellular conditions, effectively replicating the intracellular RNA folding landscape. This system is integrated with microfluidic droplet sorting to evolve RNA aptamers. Through this approach, an RNA aptamer is engineered with improved fluorescence activity by exploring the chemical fitness landscape under biomimetic conditions. The enhanced RNA aptamer named eBroccoli has increased fluorescence intensity and thermal stability, both in vitro and in vivo in bacterial and mammalian cells. In mammalian cell culture conditions, a fluorescence improvement of 3.9‐times is observed and biological thermal stability up to 45 °C is observed in bacterial systems. eBroccoli enable real‐time visualization of nanoscale stress granule formation in mammalian cells during heat shock at 42 °C. By introducing the concept of “biomimetic equivalence” based on RNA folding, the platform offers a simple yet effective strategy to mimic intracellular complexity in evolution‐based engineering.

## Graphical abstract

Microfluidic‐based directed evolution is a powerful tool for selection of biopolymers, however they struggle in transferability to intracellular environments. The “biomimetic equivalence” principle provides a versatile and practical framework for developing biopolymers that function effectively in cells using a cell‐free approach. This strategy is demonstrated by enhancing an RNA aptamer for tracking heat‐induced stress granule formation in live cells.

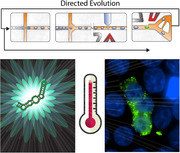

## 1 Introduction

Biopolymers serve as unique scaffolds in combinatorial chemistry, utilizing unannotated libraries that surpass conventional annotated libraries in size. They have diverse applications, from catalysis to therapeutics and diagnostics. Directed evolution of biopolymers, including RNA, demands both function and heredity, with challenges arising from two main aspects. First, sequence space complexity hinders experimental ergodicity, and despite deep‐learning‐assisted computational design,^[^ ^1^, ^2^ ^]^ routine identification of high‐efficiency sequences remains elusive. Second, the scarcity of methodologies for creating distinct microenvironments for biopolymer folding and function presents a barrier to sequence effectiveness. Emerging protocell models, both membranized and non‐membranized compartments,^[^ ^3^, ^4^ ^]^ have yet to be fully integrated. These obstacles underline the contrast between the sequence space's vastness and the extreme rarity of life‐supporting sequences, emphasizing the need for more efficient evolution principles in biopolymer engineering.

Aptamers are short ssDNA or RNA sequences capable of specific, high affinity binding to targets as diverse as whole cells, proteins, peptides, small molecules, and metal ions. They are used as recognition molecules for therapeutics and diagnostics. Aptamers are functional biomolecules with intrinsic heredity, the characteristic required for directed evolution. One application of aptamers is as light‐up fluorescence pairs. These pairs consist of an RNA sequence that binds and activates the fluorescence of a small‐molecule fluorogen. Fluorogens, such as the green fluorescent protein (GFP) chromophore‐based DFHBI,^[^ ^5^ ^]^ have advantages such as cell‐membrane permeability, low toxicity, and low background fluorescence, making them well suited for live‐cell imaging but with minimal perturbation to RNA functions. RNA aptamers, such as Spinach^[^ ^5^, ^6^ ^]^ and Broccoli,^[^ ^7^ ^]^ specifically bind and activate the fluorescence of DFHBI and have been utilized as RNA imaging tools.^[^ ^8^, ^9^ ^]^ Additional chromophores and corresponding RNA aptamers such as Pepper,^[^ ^10^ ^]^ Chilli,^[^ ^11^ ^]^ Mango,^[^ ^12^, ^13^ ^]^ Okra^[^ ^14^ ^]^ and RhoBlast^[^ ^15^, ^16^ ^]^ have increased the spectral range of light‐up fluorogenic RNA aptamers.

A significant challenge in using fluorogenic RNA aptamers for live‐cell imaging is the impaired fluorescence compared to in vitro assays,^[^ ^17^ ^]^ mainly due to the lower RNA folding efficiency in response to pressures of suboptimal folding conditions in physiological environments. As many aptamers are selected within refined buffer at room temperature, their performance at physiological temperature is not guaranteed. This challenge becomes particularly critical for RNA imaging in biomolecular condensates formed via liquid‐liquid phase separation,^[^ ^18^ ^]^ which have unique microenvironments including decreased polarity,^[^ ^19^, ^20^ ^]^ complex architectures,^[^ ^21^, ^22^ ^]^ pH gradients,^[^ ^23^ ^]^ insufficient Mg^2+^ partitioning,^[^ ^24^ ^]^ and pressures on RNA folding landscapes ^[^ ^25^ ^]^ including RNA structural stability^[^ ^25^, ^26^ ^]^ and ribozyme activities.^[^ ^27^, ^28^ ^]^ RNA hybridization and folding kinetics have been shown to decrease within in vitro condensates. Coacervates of synthetic RNA and recombinant CAPRIN1 protein exhibit decreased k_on_ and increased k_off_ rates of RNA hybridization when compared to buffer. This results in impaired RNA folding within the coacervates.^[^ ^29^ ^]^ Furthermore, increased recognition of the importance of RNA condensates, such as heat‐shock granules formed in response to temperature stress (up to 46 °C),^[^ ^30^ ^]^ necessitates the development of brighter, thermally stable RNA aptamer‐fluorogen pairs. Creating compartments that mimic intracellular environments with high pressures on RNA folding landscapes is crucial for selecting RNA aptamers suitable for physiological conditions. While synthetic protocells based on complex coacervates and/or liposomes excel at emulating intracellular conditions,^[^ ^3^ ^]^ their integration into high‐throughput screening systems remains challenging, due to interference between interaction networks underlying biomimetic compartment assembly and RNA library generation.^[^ ^31^ ^]^

In this work, we introduced a biomimetic equivalence principle for RNA folding landscapes, integrating it with directed evolution to select highly fluorescent and thermally stable fluorogenic RNA aptamers for cellular imaging. We demonstrated that heating water‐oil droplet emulsions exerts pressures on RNA folding landscapes akin to intracellular conditions. Utilizing high‐throughput droplet microfluidics, we therefore applied temperature as a selection pressure to simulate intracellular environments, enabling directed evolution of Broccoli aptamers. Error prone PCR was used to create the initial Broccoli tRNA(BroccoliT) variant library. We performed five rounds of high‐throughput droplet microfluidics at increasing temperatures with error‐prone PCR between selection cycles to evolve and isolate RNA aptamers capable of fluorescence activation of the fluorogen. Our best aptamer, termed as eBroccoli, had a fluorescence increase of 5.8‐fold at 45 °C, 3.7‐fold at 37 °C, and 1.6‐fold at 25 °C, when compared to the original Broccoli aptamer. This increased thermal stability translated into increased fluorescence brightness when imaging tagged RNA in mammalian cells. More importantly, with eBroccoli, we demonstrate the first attempt at fluorogenic RNA aptamer‐based imaging within temperature‐induced stress granules up to 42 °C. Our results emphasize the considerable potential of using cellular condensate‐mimetic conditions as a selection pressure to evolve new fluorogenic RNA aptamers.

## 2 Results

### 2.1 Microdroplets as an Intracellular Mimetic Environment

**Figure 1:**
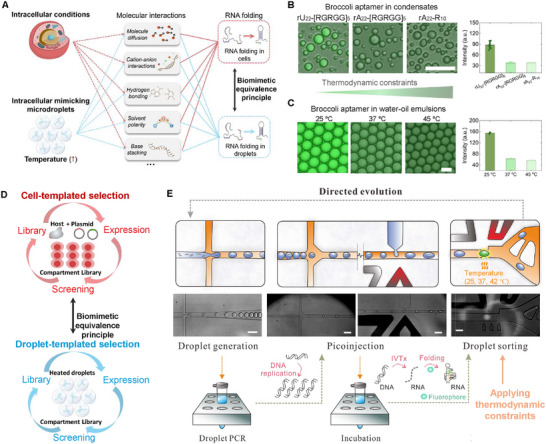
The biomimetic equivalence principle for constructing an intracellular‐like environment using heated water‐oil emulsions. A) The influence of cellular microenvironments and heated microdroplet environments on the molecular interactions that dictate RNA folding dynamics. B) The fluorescence of the RNA Broccoli aptamer in three distinct RNA‐peptide condensate types (scale bar: 20 µm, *n* = 15). C) The fluorescence of the RNA Broccoli aptamer in water‐oil emulsions exposed to three different temperature conditions (scale bar: 20 µm, *n* = 6). D) Comparison between cell‐templated and droplet‐templated selection methods for RNA aptamers. E) Schematic representation of the droplet microfluidic‐assisted directed evolution process for Broccoli aptamers. Scale bar: 100 µm.

Intracellular factors, such as macromolecular crowding, pH gradients, local micropolarity, and metabolites, influence molecular interactions that govern RNA folding in cells, including electrostatic interactions, hydrogen bonding, and base stacking (**Figure** **1**A). It has been suggested that some light‐up fluorescence aptamers had strong in vitro performance but performed poorly in vivo, as in vivo conditions apply greater pressure on RNA folding kinetics.^[^ ^32^ ^]^ To better mimic in vivo RNA folding, we established a biomimetic equivalence principle using heated microdroplets as compartments to apply similar pressure to RNA folding kinetics. We introduced the RNA aptamer Broccoli into various compartments and used its fluorescence as an indicator to characterize RNA folding pressures (Figure 1A).

To assess the effect of the microenvironment on RNA aptamer folding, we prepared three distinct RNA‐peptide condensates: rU_22_‐[RGRGG]_5_, rA_22_‐[RGRGG]_5_, and rA_22_‐R10, with increasing RNA‐peptide interaction strength.^[^ ^33^ ^]^ The increased interaction strength results in the inhibition of guest nucleic acid partitioning and folding,^[^ ^25^, ^34^ ^]^ As shown in Figure 1B, the fluorescence intensity of the RNA Broccoli aptamer within rA_22_‐[RGRGG]_5_ and rA_22_‐R10 is significantly lower than in rU_22_‐[RGRGG]_5_. These results demonstrate that the microenvironment within biomimetic compartments affects RNA aptamer performance, with fluorescence decreasing as thermodynamic constraints within the compartments increase. To mimic this behavior, we encapsulated RNA Broccoli aptamers within microfluidic water‐oil emulsions at different temperatures: 25, 37, and 45 °C. Due to high‐temperature‐induced pressures on RNA folding, the fluorescence of emulsions at 25 °C is much higher than that at 37 and 45 °C (Figure 1C), displaying a pattern similar to aptamer fluorescence in biomolecular condensates in response to thermodynamic constraints. We concluded that heated microdroplets can effectively mimic intracellular conditions that exert pressure on RNA aptamer folding.

### 2.2 Droplet Assisted Evolution and Screening of New Broccoli sequences

Next, we applied the biomimetic equivalence principle to the evolution and selection of new RNA aptamers with enhanced in vivo performance. Since heated microdroplets have been demonstrated to exert similar pressures on RNA folding as those found in intracellular conditions, we utilized these droplets under varying temperatures as a substitute for cell‐templated selections (Figure 1D).

To evaluate the evolution performance of our droplet‐assisted aptamer selection, the droplet fluorescence intensity is recorded during the fluorescence activated droplet sorting (FADS) (Figure 1E). The initial aptamer library, obtained through the mutagenesis on the BroccoliT template, showed relatively low droplet brightness after the in vitro transcription (Figure S1C, Supporting Information). After the 1st round of the selection, recovered templates undergo mutagenesis before seeding the 2nd round of the selection. Compared with the 1st round of the selection, droplet brightness has a significant increase in the 2nd round (Figure S1A,C, Supporting Information). By comparing the droplet fluorescence intensity histograms from round 1 and round 2, the maximum droplet fluorescence intensity increases more than twofold (Figure S1B, Supporting Information), which suggests an effective evolution of aptamer species in the library. After the initial two rounds of the selection, the experimental temperature was increased from 25 °C to 37 °C in the round 3 selection, then further increased to 42 °C in the round 4 and round 5 selection (Figure S1D, Supporting Information). This gradual increase in temperature over the selection rounds was used to avoid population collapse from overzealous selection pressure. As we used mutation for pool diversification, evolution could occur in a stepwise manner over multiple selection rounds.

From droplet fluorescence intensity histogram data (Figure S1, Supporting Information), the maximum droplet fluorescence intensity from round 3 to round 5 stays in the same level with that of round 2, indicating the existence of potential aptamer species that have comparable fluorescence intensity at higher thermal stability. Comparing the fluorescence histogram in round 4 and round 5 (Figure S1B, Supporting Information), no significant change is identified. The averaged droplet fluorescence intensity of the most fluorescent 1% droplets in each round are plotted in Figure S1D (Supporting Information), which also suggests a significant improvement in droplet brightness from round 1 to round 2. The fraction of droplets with the maximum fluorescence decreases from round 2 to round 3 and round 4. This is not surprising as the selection temperature increases over these rounds and most likely disrupts the folding of most aptamers in the selection pool. Through the implementation of high temperature selection pressure, we evolved and screened aptamers with improved thermal stability. The fluorescence change from round 4 to round 5 was negligible, so we sequenced the aptamer pools and characterized the RNA aptamers. Similar microfluidic selection systems have been applied to isolate new RNA aptamers like iSpinach and Mango. To our best knowledge, this is the first microfluidic‐assisted Broccoli RNA aptamer evolution and screening reported.

### 2.3 Characterization of New eBroccoli Aptamer Sequences

Using sequencing, many broccoli variants are identified, giving a picture of the broccoli aptamer family sequence space. Interestingly, some of the most abundant sequences showed little homology to the original broccoli sequence and had no measurable fluorescence upon DFHBI‐1T incubation. Similar observations have been made in other selections and are deemed to be the result of PCR bias.^[^ ^35^, ^36^ ^]^ Through clustering, we isolated the broccoli aptamer family and characterized highly abundant individuals.

**Figure 2:**
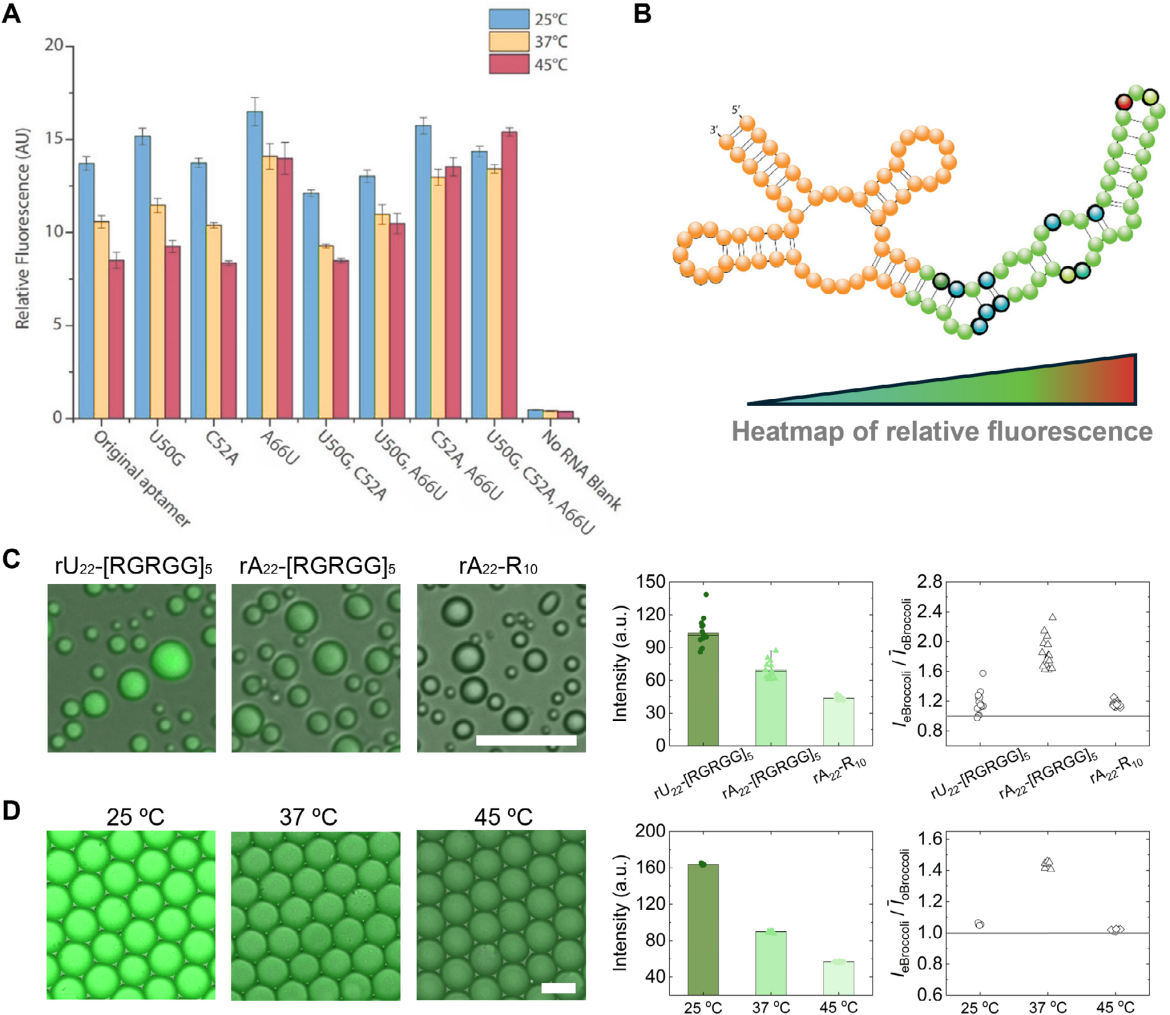
Isolation of eBroccoli aptamer sequence and fluorescence characterization within different compartments. A) In vitro fluorescence characterization of single, double and triple BroccoliT mutant variants. The fluorescence intensities of the purified RNA variants with DFHBI‐1T was measured in a 384‐well plate at the temperatures 25, 37, and 45 °C. The temperature dependent change in fluorescence signal indicates the stability of each variant. Data presented as mean ± SEM (*n* = 3). B) Broccoli aptamer family mutation sites overlayed onto secondary structure. Mutation sites are highlighted in bold and single point mutation variant fluorescence is denoted according to the heatmap. Many point mutations are at functionally important sites on the aptamer sequence. C) The fluorescence of the triple mutation BroccoliT (eBroccoli) in three distinct RNA‐peptide condensate types, as well as their fluorescence relative to the original Broccoli (oBroccoli) aptamer (scale bar: 20 µm, *n* = 15). D) The fluorescence of eBroccoli in water‐oil emulsions exposed to three different temperature conditions, as well as their fluorescence relative to the oBroccoli aptamer (scale bar: 20 µm, *n* = 6).

The broccoli aptamer family included twelve mutational hotspot sites of interest (**Figure** **2**A,B; Figure S2, Supporting Information). In vitro fluorescent activity measurement of the twelve single point mutant variants (Figure S3, Supporting Information) screened three mutations U50G, C52A, and A66U, that are of particular interest. Analysis of synergistic effect of these mutations (Table S1, smll202409911‐sup‐0002‐VideoS1.mp4) at varying temperatures revealed the mutational combinations that lead to a greater florescent heat tolerance (Figure 2A,B). The triple mutant U50G, C52A, and A66U (Figure 2A,B) does not appear to suffer from any fluorescence loss even at the higher temperature of 45 °C (Figure S4, Supporting Information). In particular, when the triple mutant was encapsulated within RNA‐peptide condensates, a significant fluorescence enhancement was observed compared to the original Broccoli aptamer, as depicted in Figure 2C. These findings indicate that the triple mutant exhibits improved folding kinetics within biomolecular condensates. The observed enhancement of droplet fluorescence under different temperature conditions (Figure 2D) further supports this conclusion.

### 2.4 In vivo Applications of eBroccoliT

**Figure 3:**
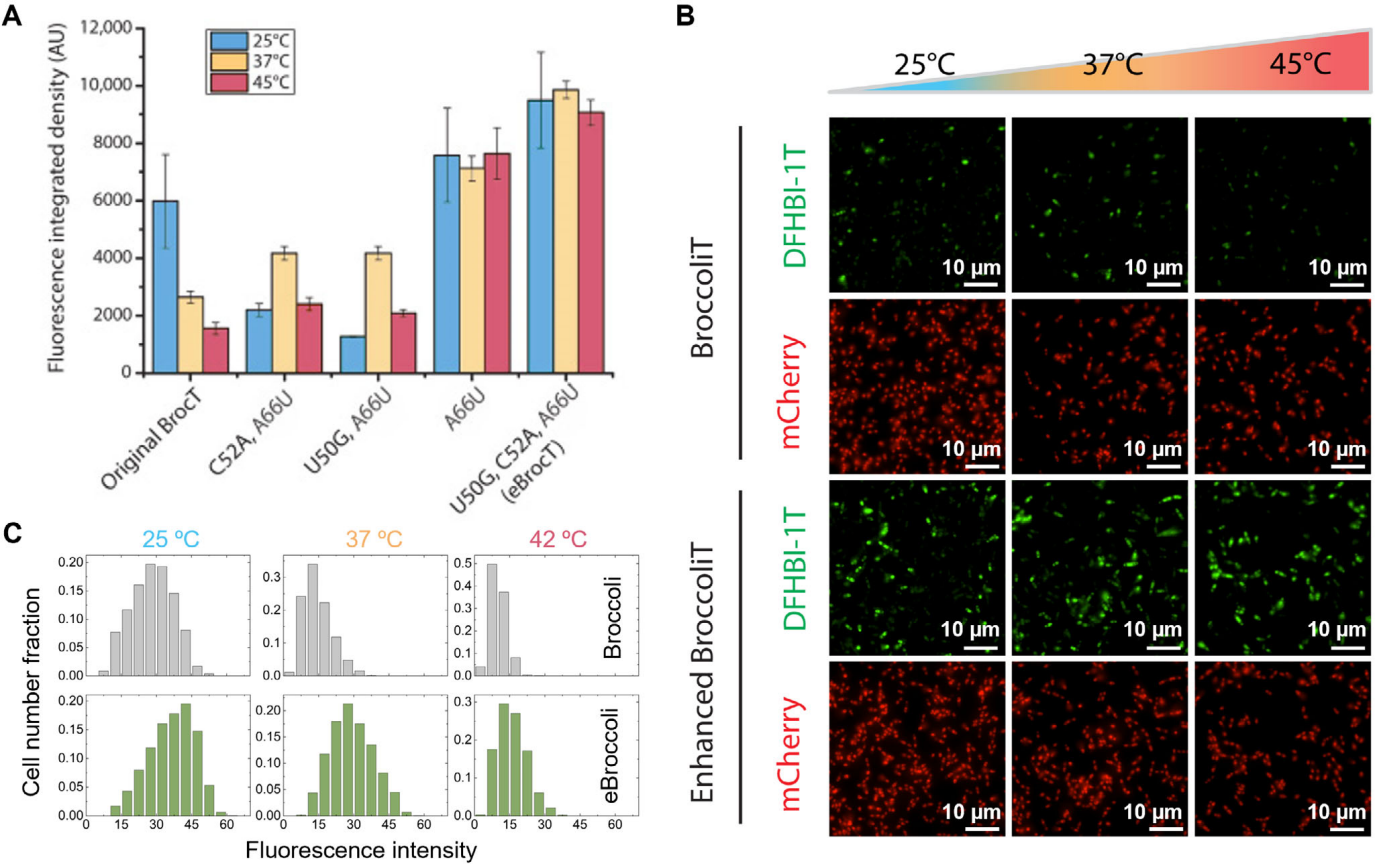
eBroccoli aptamer performance in *E. coli*. A) Quantification of microscope image fluorescent signal for *E. coli* co‐expressing broccoli variants and mCherry. Data was collected at 25, 37, and 45 °C and DFHBI‐1T fluorescent signal was normalized to mCherry signal. Data presented as mean ± SEM (*n* = 5). B) Microscope images of *E. coli* expressing original BroccoliT (oBroccoli, upper) and eBroccoliT (lower) over increasing temperatures. mCherry was co‐expressed as a normalizing control. C) Distribution of cellular fluorescence levels for BroccoliT (grey) and eBroccoliT (green) at 25, 37, and 45 °C. Widefield fluorescence images were taken with five replicates per group using 100 ms exposure with 100x lens 488 nm laser with the 512/30 nm filter for DFHBI and the 561 nm laser with 592/30 nm filter for mCherry.

To examine the in vivo fluorescence activity of our aptamers we expressed them in bacteria. Plasmids were constructed that co‐express each eBroccoliT variant with mCherry as a normalizing control for imaging (Figure S6, Supporting Information). Imaging of bacteria expressing the BroccoliT variants (**Figure** **3**B) revealed that their in vivo performance matched their strong in vitro performance. The triple mutant eBroccoliT has a fluorescence increase of 1.6‐fold at 25 °C, 3.7‐fold at 37 °C, and 5.8‐fold at 45 °C when compared to the original BroccoliT aptamer (Figure 3A; Figure S5, Supporting Information). The thermostability of eBroccoliT at the biologically relevant temperature of 37 °C (Figure 3C) is desirable for cell culture experiments. Similar to the in vitro data, the in vivo data shows eBroccoliT does not appear to suffer from any fluorescence loss even at the higher temperature of 45 °C.

### 2.5 Application of eBroccoli in Mammalian Cell Culture

**Figure 4:**
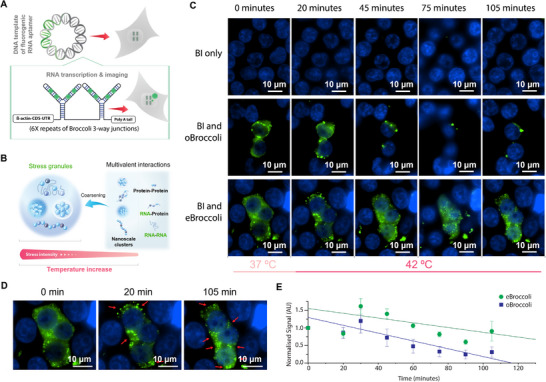
eBroccoli aptamer performance in mammalian cell culture: observation of heat shock‐induced stress granule formation. A) Transfection of plasmid containing transcript template with the design of the β‐actin‐CDS‐UTR broccoli tagged transcript. Plasmids coding the transcript were used in both the original broccoli and enhanced broccoli versions. B) Representative examples of heat shock induced stress granule formation for BI fluorogen only, oBroccoli and BI, and eBroccoli and BI. Cells were initially imaged at 37 °C before incubation at 42 °C and imaging over 105 min. C) Schematic of heat stress granule formation. D) Zoomed in time course of stress granule formation, imaged with eBroccoli and BI. Green fluorescent puncta are highlighted with red arrows. E) Comparison of the thermal stability of eBroccoli and oBroccoli over time during heat shock conditions. Live cell time course images were taken with the 405 nm and 488 nm lasers were set at 5% and 10% of maximum power captured under Airyscan mode. Data presented as mean ± SEM (*n* = 5).

The main desired application of light‐up fluorescence aptamers is for analysis of RNA in mammalian cells. To investigate stability and fluorescence enhancements of eBroccoli in mammalian cells we utilized the broccoli 3‐way junction design ^[^ ^17^ ^]^ (**Figure** **4**A). In this design the unit is made up of a three‐way junction connecting two diametric broccoli sections, creating 4 fluorogen binding pockets per unit. Our β‐actin‐CDS‐UTR broccoli tagged transcript contained 6 broccoli 3‐way junction units, totaling 24 fluorogen binding pockets per transcript (Figure 4A). The oBroccoli and eBroccoli transcripts were each cloned into a pcDNA3.1(+) plasmid that is used for cell transfection. When compared to the original broccoli sequence in HEK293 cells at 37 °C, eBroccoli had 3.9‐times the fluorescence enhancement (Figure S8, Supporting Information). This result is consistent with the bacterial fluorescence analysis (Figure 3A; Figure S5, Supporting Information) and the fluorescence light‐up melt curve assay (Figure S9, Supporting Information) which showed that eBroccoli's T_m_ (37.0 °C) is 2.5 °C higher than oBroccoli's (34.5 °C).

To utilise eBroccoli's thermal stability experimentally, we observed heat‐induced stress granule formation (Figure 4B). By performing time‐lapse microscopy of both the β‐actin‐CDS‐UTR eBroccoli and β‐actin‐CDS‐UTR oBroccoli plasmids transfected HEK293 cells over increasing temperature, we compared the utility of oBroccoli and eBroccoli at observing heat induced stress granules. At 37 °C, green fluorescent puncta were observed throughout the cell. Upon temperature increase to 42 °C, the green fluorescent puncta aggregated into larger foci with the morphology of stress granules (Figure 4C). The fluorescence signal observed for eBroccoli was greater and had increased thermal stability (Figure 4D,E).

## 3 Conclusion

To demonstrate our biomimetic equivalence principal, we introduced the innovative step of using temperature and low salt concentration as an evolutionary selection pressure in the droplet microfluidic screening of Broccoli light‐up aptamer variants. Our biomimetic selection pressure resulted in the identification of the triple mutant eBroccoli with an in vivo fluorescence increase of 1.6‐fold at 25 °C, 3.7‐fold at 37 °C, and 5.8‐fold at 45 °C when compared to the original Broccoli aptamer.

Microfluidic droplet selections have been used to improve fluorescence light‐up aptamers previously. iSpinach was isolated through microfluidic droplet selection and had increased fluorescence activity relative to its Spinach derivative but only in vitro.^[^ ^37^ ^]^ Here, eBroccoli shows both in vitro and in vivo enhanced fluorescence activity demonstrating the utility of our biomimetic equivalence principle. iSpinach was selected in a relatively high salt concentration (≈60 mm vs our 5 mm) to enhance aptamer folding. Our biomimetic selection methodology applied high selective pressure on RNA folding. Cell macromolecular crowding, pH gradients, local micropolarity, and metabolites all influence molecular interactions that govern RNA folding, including electrostatic interactions, hydrogen bonding, and base stacking. To select for RNA folding, we mimic those folding microenvironments with heat and lower salt concentration. Although intracellular potassium levels are relatively high (≈140 mM), the net effect of macromolecular crowding, pH gradients, local micropolarity, and metabolites result in inhibition of RNA folding.

Mango IV was isolated through a competitive ligand binding microfluidic selection process and showed increased fluorescence activity relative to its Mango I derivative.^[^ ^13^ ^]^ Mango IV has been used for imaging small non‐coding RNAs in mammalian cells. To our knowledge eBroccoli is the only microfluidic selected, GFP based chromophore binding aptamer that has enhanced in vivo activity. Furthermore, the eBroccoli light‐up aptamer displayed enhanced fluorescence signal and thermostability in mammalian cells. Nanoscale green fluorescent puncta (Figure S8, Supporting Information) were easily observed with similar morphologies to other techniques such as the MS2 system.^[^ ^38^, ^39^ ^]^

The MS2 system utilizes an RNA hairpin that recognizes a fluorescent protein as the probe. Although this system has given insight into RNA synthesis, such as the observation of discontinuous transcription bursts separated by irregular intervals,^[^ ^40^, ^41^, ^42^ ^]^ it has some inherent limitations. The MS2 system is reliant on an MS2 fusion protein as the fluorescence probe. This protein is large and is chemically different to RNA, potentially interfering with tagged mRNA function, movement, and localization. In addition, the MS2 protein contains a nuclear localization signal^[^ ^43^ ^]^ that affects the location of the RNA complex, accumulating the MS2 fusion protein in the nucleus and potentially confounding mRNA localization. Light‐up aptamer/fluorogen mRNA tagging systems incorporate RNA and small molecule fluorogens so the effect on mRNA function, movement, and localization are minimized.

Application of the biomimetic equivalence principal to microfluidic droplet‐based selection enables the selection of diverse properties compatible with the intracellular micro environment. Using microfluidic selection, nucleic acids with other attributes can be selected including nuclease activity,^[^ ^44^, ^45^ ^]^ ligase activity,^[^ ^46^ ^]^ and catalysis.^[^ ^47^ ^]^ Similarly, using microfluidic selection, proteins have been selected for many catalytic applications such as hydrolase,^[^ ^48^ ^]^ oxidases,^[^ ^49^ ^]^ aldolase ^[^ ^50^ ^]^ and polymerase.^[^ ^51^ ^]^ As many proteins and their biological processes are associated with biomolecular condensates and the altered folding environment, the biomimetic equivalence principal would be useful for their directed evolution. Adoption of the of the biomimetic equivalence principal to protein directed evolution would similarly require low salt and high temperature conditions as protein folding is enhanced by salt. The great benefit of microfluidics is that molecular evolution can be guided by a wide variety of biologically incompatible selection pressures, such as by high temperature and low salt, in this work.

Due to their unique physicochemical properties, water‐oil emulsions, along with their interfaces, have been widely proposed as templates for protocell construction and as model systems for biocatalysis.^[^ ^52^, ^53^, ^54^ ^]^ These results offered inspiration in constructing a cell‐mimicking environment within microfluidics to screen new RNA aptamers.

Our microfluidic selection workflow is divided among three microfluidic chips (droplet generation, pico‐injection, and droplet sorting) with off chip processing in between these steps (thermal cycling, transcription, sequence recovery PCR). The integration of off‐chip procedures such as thermal cycling and transcription is feasible^[^ ^55^ ^]^ and would significantly enhance the advantages of microfluidics, including increased efficiency, automation, and parallel processing capabilities. However, this integration introduces challenges related to device fabrication and setup complexity, thermal management and higher maintenance requirements during experiments.

Our biomimetic equivalence principal microfluidic droplet‐based selection system successfully evolved a thermostable, bright, light‐up fluorescent aptamer. The eBroccoli aptamer will be a useful robust tool for RNA imaging, particularly where cellular imaging is required under higher temperature stress conditions. Perhaps future iterations of the biomimetic equivalence principal could incorporate liquid‐liquid phase separation condensates into microfluidic droplets to better mimic the intracellular environment. Indeed, condensates have been incorporated into microfluidic droplets to accurately assess partition coefficients of various molecules with diverse coacervate chemistries.^[^ ^56^ ^]^ Applying evolutionary selection pressures to such systems could extend the repertoire of directed evolution.

## 4 Experimental Section

### Microfluidic Aptamer Selection Workflow

The workflow of the droplet‐based microfluidic selection (Figure 1A) consists of the PCR mix including 1 pM Broccoli variant library being dispersed into 20 pL monodispersed microdroplets. Each droplet contains ≈0.2 DNA template copies and based on a Poisson distribution, this guarantees that more than 98% droplets contain no more than 1 DNA template, ensuring monoclonal droplets. Next, these droplets are collected and undergo 40 PCR cycles. The PCR droplets are reinjected and spaced into a picoinjection device. Using an electric field induced instability on the interface, transcription mixture containing DFHBI‐1T is injected into PCR droplets as they pass through the picoinjection junction. The picoinjected droplets are then collected and incubated for 4 h at 37 °C to allow transcription of RNA aptamers and fluorogen binding. The droplets are introduced into a sorting device for FADS under the temperature of 25, 37 or 45 °C. Droplets with higher fluorescence intensity (≈1%) are sorted and collected for DNA recovery. Error prone PCR is used to diversify aptamers pools in between selection rounds.

### Library Design/Generation

The library was generated using mutagenic PCR. The BroccoliT sequence (GCCCGGATAGCTCAGTCGGTAGAGCAGCGGAGACGGTCGGGTCCAGATATTCGTATCTGTCGAGTAGAGTGTGGGCTCCGCGGGTCCAGGGTTCAAGTCCCTGTTCGGGCGCCA) was flanked with the forward primer – promoter – adapter sequence (ATACGAGCTTGTTCAATATAATACGACTCACTATAGGAAGACGTAG CAAG) and the reverse primer reverse complement sequence (TGATAGTAAGAGCAATC). The initial library was generated by mutagenic PCR.

### PCR Optimization

In order to perform the optimal number of PCR cycles, a process of PCR optimization is performed. A bulk PCR reaction of 100 µl is made and multiple 2 µl aliquots of this PCR reaction are taken according to the desired PCR optimisation range. The multiple 2 µl aliquots undergo varying amounts of thermal cycling before native PAGE analysis. PAGE analysis reveals the optimal number of PCR cycles required for the sample in regard to yielding a strong PCR product band, without the presence of non‐specific amplicon. The appropriate number of PCR cycles are then performed on the remaining bulk PCR sample.

### Mutagenic PCR

Mutagenic PCR is facilitated using the promiscuous dNTP analogs 8‐oxo‐dGTP and dPTP.^[^ ^57^ ^]^ This process involves 2 steps: 1) Using PCR to insert these promiscuous dNTP analogs randomly in the template sequence and 2) Using a separate PCR reaction to replace the promiscuous dNTP analogs with a random natural dNTP.

### Inserting Promiscuous dNTP Analogs

PCR reaction is made containing 1X Taq polymerase buffer (NEB), 50 fmol of DNA template, 400 nM of forward and reverse primers, 100 µm each natural dNTP, 200 µm 8‐oxo‐dGTP, 200 µm dPTP, and 5 units of Taq polymerase (NEB) in a 100 µL reaction. The PCR mix underwent thermal cycling using the following program: one cycle of 96 °C 1 min followed by 10 to 20 cycles (according to PCR optimisation protocol) of 96 °C for 30 s, 52 °C for 30 s, and 72 °C for 45 s, followed by one cycle of 72 °C for 20 s and hold at 4 °C.

### Removing Promiscuous dNTP analogs

A PCR reaction is made containing 1X Taq polymerase buffer (NEB), 5 pmol of DNA template, 400 nm of forward and reverse primers, 300 µm each natural dNTP, and 5 units of Taq polymerase (NEB) in a 100 µL reaction. The PCR mix underwent thermal cycling using the following program: one cycle of 96 °C 1 min followed by 10 to 20 cycles (according to PCR optimization protocol) of 96 °C for 30 s, 52 °C for 30 s, and 72 °C for 45 sec, followed by one cycle of 72 °C for 20 sec and hold at 4 °C.

### Microfluidic Droplet Generation and PCR

A PCR reaction was made containing 1X PFU polymerase buffer (Promega), 3.2 µg mL^−1^ Yeast RNA, 32 fmol of DNA template, 800 nm of forward and reverse primers, 300 µm each natural dNTP, 0.1% Pluronic acid, 670 µg mL^−1^ 70 kDa dextran and 5 units of PFU polymerase (Promega) in a 100 µL reaction. The PCR mixture is withdrawn at 2000 µL/hr using a syringe pump (neMESYS low pressure module, Cetoni GmbH) into a polytetrafluoroethylene tubing (BB311‐24, Scientific Commodities Inc.), which is connected to a 1 mL glass syringe (Hamilton) preloaded with FC‐40 fluorinated oil (51142‐49‐5, Sigma‐Aldrich). The PCR mixture is then injected into a flow‐focusing droplet generator, carried by outer oil phase containing HFE‐7500 fluorinated oil (3M) supplemented with 5% (w/w) fluorosurfactant (Ran biotechnologies, Inc.). The droplets are generated at ≈2100 Hz and collected in a 0.2 mL PCR tube, where the bottom oil layer is replaced with FC‐40 fluorinated oil with 5% (w/w) fluorosurfactant to improve thermal stability. The droplets are thermal cycled (G‐storm) with 98 °C for 3 mins, followed by 40 cycles with 2 °C s^−1^ ramp rates of 98 °C for 10 s, 52 °C for 20 s, and 72 °C for 20 s, ended by a hold at 4 °C.

In the method, droplet size plays a crucial role in the encapsulation efficiency of the DNA template. For example, a DNA template buffer with a concentration of 1 pm was emulsified, resulting in a 20 pL droplet size. This corresponds to an average of 0.2 copies of the DNA template within each droplet.

According to the Poisson distribution:

(1) $$P\left(X=k\right)=\frac{e^{-\lambda }\lambda^{k}}{k!}$$

where (λ  =  0.2),

(2) $$P\left(X=0\right)+P\left(X=1\right)=e^{-0.2}+0.2\times e^{-0.2}=0.9825$$

This encapsulation ensures that 98.25% of the droplets contain no more than 1 copy of the DNA template. This facilitates single‐molecule encapsulation, ensuring that each droplet carries at most one type of DNA or RNA after PCR and transcription.

If the droplet size is larger, for example, 40 pL, on average, each droplet will encapsulate 0.4 copies of DNA templates. The proportion of droplets containing no more than 1 DNA template decreases to 93.84%. This increase in droplet size results in ≈6.2% of the droplets containing at least two types of DNA templates, which may ultimately lead to false positive signals during droplet sorting, and thereby lowering sorting accuracy and efficiency. Conversely, if the droplet size is smaller, such as 10 pL, the proportion of droplets containing no more than 1 DNA template increases to 99.53%. However, this reduction in droplet size doubles the total number of droplets to be screened, thereby doubling the time required for the droplet sorting module. In conclusion, the droplet size is chosen as a balance between single‐molecule encapsulation efficiency and time cost in the experiments.

### Picoinjection and Transcription

PCR droplets are reinjected into a picoinjection device and spaced with HFE‐7500 fluorinated oil supplemented with 1% (w/w) fluorosurfactant. The droplets are merged at the picoinjection junction with the in vitro transcription (IVT) mixture containing containing 1X transcription buffer (Epicentre), 9 mm each NTP, 10 mm DTT, 35 µg mL^−1^ 70 kDa dextran, 3.5% Pluronic acid, 200 µm DFHBI‐1T (Lucerna), 1 mm Spermidine, Inorganic 0.1 units thermostable PPase, 0.5 units SuperRNAse•In RNase Inhibitor (Thermo Fisher Scientific), 10 µl AmpliScribe T7‐Flash Enzyme Solution (Epicentre) in a 100 µL reaction. Injection occurs by applying a 10 kHz square wave at 400 V with a high voltage amplifier (5/80, Trek) to the electrodes on‐chip. The injection rate is ≈500 Hz. The droplets are collected in a 0.2 mL PCR tube, and the bottom oil layer is replaced with FC‐40 fluorinated oil with 5% (w/w) fluorosurfactant. The droplets are incubated at 37 °C for 1 hr. RNA transcription occurs during the incubation and transforms the DFHBI fluorogenic substrate into a fluorescent state.

### Microfluidic Sorting

Incubated droplets are reinjected into droplet sorter and spaced with surfactant free HFE‐7500 fluorinated oil. The temperature was calibrated using a Platinum resistance thermometer (Pt100Ω) with an accuracy within 0.1 °C (Figures S10 and S11; Video S1, Supporting Information). These temperatures (25, 37, and 45 °C) are below those typically used in microfluidic‐based digital LAMP,^[^ ^58^ ^]^ where bubble formation via heating is negligible. The droplets are reinjected at a frequency of ≈250 Hz by adjusting the flow rates. Consequently, screening one million droplets requires ≈ 1.11 h in the sorting module. Approximately 2 million droplets were sorted for each microfluidic selection round. Droplets with 1% most green fluorescence (concentration of the fluorogenic product) are gated, and sorted into the positive channel. The bright droplets are deflected into the positive channel by applying a 30 kHz square wave at 1000 V with a high voltage amplifier. The optical setup and programs are described in the previous work.^[^ ^59^ ^]^ The sorted droplets are recovered by mixing with 1H,1H,2H,2H‐perfluoro‐1‐octanol (370533, Sigma‐Aldrich) followed by brief centrifugation. The upper aqueous phase is pipetted to a new tube for downstream DNA sequencing. To reduce droplet sorting errors caused by polydisperse droplet sizes the NOVAsort strategy may be used.^[^ ^60^ ^]^ Investigations on precisely recognizing polydisperse droplets using artificial intelligence also hold promise for further reducing the sorting errors and increasing the sorting rate.^[^ ^61^ ^]^

### Sequencing of SELEX Pools

High throughput sequencing was used to analyze the microfluidic selection results. Six aptamer pools (initial library, round 1 to 5) underwent PCR with unique barcoded primers before agarose gel purification, silica spin column purification. The samples were submitted to Novogene for sequencing and 3GB of paired end 150 bp data was sequenced on the NovaSeq PE150 platform.

### Fluorescence Screening Assay

For the in vitro screening of Broccoli variants, a microplate assay was used. For each broccoli variant, 10 µL of 0.5 µm DNA template was mixed with 10 µL of transcription mix (AmpliScribe T7‐Flashkit, Epicentre) with 40 µm of DFHBI‐1T. The samples were pipetted onto a Corning 384 well microplate and fluorescence was measured at an excitation of 470 nm and emission of 505 nm over 2 h at 37 °C on a microplate reader (Varioskan Flash, Thermo Scientific).

### RNA‐Peptide Condensate Assay

RNA‐peptide condensates were prepared based on the concentration of the charged residues, with a charge concentration of [e‐] = [e+] = 5 mm. In the aqueous mixture, each arginine residue contributes one positive charge, and each RNA phosphate group contributes one negative charge. For encapsulation of RNA aptamers, a mixture of 5 µm Cy5‐labeled Broccoli or Cy5‐labeled eBroccoli aptamer and RNA oligo (U_22_ or A_22_) was prepared in a buffer containing 200 mm DFHBI‐1T, 100 mm KCl, 1 mm MgCl_2_, and 40 mM Tris (pH 7.5). The mixture was renatured at 95 °C for 3 min, cooled to room temperature, and incubated for 30 min. Peptide (R10 or [RGRGG]_5_) was then added to the mixture and mixed thoroughly using a pipette. The mixed samples were incubated at room temperature for 30 min before imaging with a Leica inverted microscope.

### Heated Water‐Oil Emulsion Assay

The aqueous mixture containing 30 µm RNA aptamer (eBroccoli or oBroccoli), 600 mm DFHBI‐1T, 100 mm KCl, 1 mm MgCl_2_, and 40 mm Tris (pH 7.5) is withdrawn at 2000 µL h^−1^ using a syringe pump (neMESYS low pressure module, Cetoni GmbH) into a polytetrafluoroethylene tubing (BB311‐24, Scientific Commodities Inc.), which is connected to a 1 mL glass syringe (Hamilton) preloaded with FC‐40 fluorinated oil (51142‐49‐5, Sigma‐Aldrich). The mixture is then injected into a flow‐focusing droplet generator, carried by outer oil phase containing HFE‐7500 fluorinated oil (3M) supplemented with 5% (w/w) fluorosurfactant (Ran biotechnologies, Inc.). The droplets are collected in 0.2 mL PCR tubes, where the bottom oil layer is replaced with FC‐40 fluorinated oil with 5% (w/w) fluorosurfactant to improve thermal stability. The droplets are thermal cycled (G‐storm) with 95 °C for 3 min, and cooled to room temperature before imaging with a Leica inverted microscope.

### Quantitative Fluorescence Assay

For the in vitro characterization of Broccoli variants, a microplate assay was used. RNA was prepared using the AmpliScribe T7‐Flashkit (Epicentre) and DNA template of the aptamer sequence. Following ethanol precipitation of the transcription product, the concentration of RNA was measured using the Qubit RNA HS Assay (Life Technologies, Thermo Fisher Scientific Inc.). A fluorescence assay mix was made containing 0.5 µM RNA and 10 µM DFHBI‐1T or BI in fluorescence assay buffer (20 mm Tris pH 7.5, 10 mm NaCl, 0.5 mm KCl, 0.2 mm MgCl_2_, 0.1 mm CaCl_2_). The mixture was incubated for 10 min at the desired temperature (25 °C or 37 °C or 45 °C) before the fluorescence was measured at an excitation of 470 nm and emission of 505 nm on the microplate reader (Varioskan Flash, Thermo Scientific).

### Cloning of the Plasmids used for the Aptamers Imaging in Bacterial Cells

The 9 variants plus the original BroccoliT sequence were cloned into a pET vector that coexpresses mCherry (Figure S6, Supporting Information). The pET Biotin His6 mCherry LIC cloning vector (H6‐mCherry) was a gift from Scott Gradia (Addgene plasmid # 29722; http://n2t.net/addgene:29722; RRID:Addgene_29722). These Broccoli variant/mCherry plasmids were transformed into DH5α *E. coli* strain, grown overnight for a miniprep kit (Qiagen) to isolate plasmid for later experiments. The plasmids were characterized using a restriction digest (Figure S7, Supporting Information)

### Aptamer Imaging in Bacteria

The BL21 *E.coli* strain was transformed with pET28 mCherry based expression vectors encoding the RNA aptamers and mCherry on separate transcripts. Negative control cells were transformed with the original mCherry pET plasmid. Transformed cells were plated on kanamycin LB agar plates, grown overnight at 37 °C, and single colonies were used to inoculate a kanamycin LB broth overnight culture. Five hundred µL of the resultant overnight cultures were used to inoculate 10 mL of kanamycin LB broth and grown to an OD_600_ of 0.4 (≈2 h). IPTG was added to the media to a final concentration of 1 mm and the cultures were incubated at 37 °C for 3 h. The cultures were then pelleted, resuspended in PBS and transferred to poly‐d‐lysine (ThermoFisher Scientific) coated µ‐slide 8 well dished (Ibidi) dishes. Cells were incubated in the dish for 45 min at 37 °C, washed 3 times with PBS and incubated at 37 °C for 45 min with 200 µM DFHBI‐1T in PBS. Fluorescence images were taken with a Eclypse Carl Zeiss Nikon Ti2‐E Widefield using the 100x lens 488 nm laser with the 512/30 nm filter for DFHBI and the 561 nm laser with 592/30 nm filter for mCherry.

### Cloning of the Plasmids used for the Aptamers Imaging in Mammalian Cells

The cloning of plasmids was conducted by GenScript. The original and enhanced broccoli three way junction multimers ^[^ ^17^ ^]^ were synthesized and cloned into the pcDNA3.1(+) plasmid.

### Aptamer Imaging in Mammalian Cells

HEK293T cells were seeded in Poly‐I‐lysine (Invitrogen) coated 24‐well glass bottom plate (NEST Biotech). When cells reach 70% confluency, plasmids that encoded aptamers were transfected into cells using lipofectamine 3000 (Invitrogen) following manufacturer's instruction. At 24 h after transfection, cells were incubated with BI (10 µM) for 1 h at 37 °C. Then, the medium was supplied with Hoechst 33342 (Invitrogen) at 2.5ug mL^−1^, following live cell imaging on a Carl Ziss LSM880 inverted confocal microscope. The laser 405 nm and 488 nm were set at 5% and 10% of maximum power and images were captured under Airyscan mode.

### Fluorescence Light‐Up Melt Curve Assay

To deduce and compare the T_m_ of oBroccoli and eBroccoli with respect to fluorescence activation, a qPCR thermal cycler melt curve was used. Triplicate 50 µL samples of 20 mm Tris pH 7.5, 10 mm NaCl, 0.5 mm KCl, 0.2 mm MgCl_2_, 0.1 mm CaCl_2_, 50% cell lysate (to better mimic intracellular conditions) with 2uM aptamer and 20uM DFHBI‐1T were assembled in qPCR strip tubes. A BIO‐RAD CFX Opus 96 Real‐Time PCR Instrument was used to measure the sample melt curves with the protocol.

### Cell Lysate Production

Eight million HEK293 cells were pelleted and resuspended in 500 µL of 20 mm Tris pH 7.5, 10 mm NaCl, 0.5 mM KCl, 0.2 mM MgCl_2_, 0.1 mm CaCl_2_ and sonicated on ice, 5 seconds on, 45 seconds off for 5 min. The lysed cells were centrifuged at 26000 G for 10 min at 4 °C to pellet cellular debris. The supernatant was taken and used as the stock cell lysate.

### Statistical Analysis

For each microscope image, DFHBI fluorescent signal data were normalized to DAPI fluorescence and averaged before baseline correction using the no transfection condition data. All data presentation formats are indicated in each figure legend. Sample size n is indicated in each figure legend. Statistical methods were used to assess significant differences indicated in each figure legend. Software used for statistical analysis were Excell 2016 and Origin 8.5.

## Conflict of Interest

The authors declare no conflict of interest.

## Supporting information



Supporting Information

Supplemental Video 1

## Data Availability

The data that support the findings of this study are available from the corresponding author upon reasonable request.
